# Clinical therapeutic effects of opioid analgesia for acute abdominal pain in children and young adults: A protocol for systematic review and meta-analysis

**DOI:** 10.1097/MD.0000000000026402

**Published:** 2021-08-06

**Authors:** Jie Li, Fei-Yan Hu, Guo Zhong

**Affiliations:** a Department of Emergency Surgery, Tianyou Hospital Affiliated to Wuhan University of Science & Technology; b Department of Geratology, Wuhan Red Cross Hospital, Wuhan, Hubei, China.

**Keywords:** acute abdominal pain, analgesia, efficiency, opioid, safety

## Abstract

**Background:**

Nearly 10% of all patients who visit the emergency department report severe abdominal pain. Out of these, almost one-third are not diagnosed accurately. The conventional practice to care for such inpatients involves actively managed observation and repetitive clinical assessments at regular intervals. The aim of this study is to assess the clinical therapeutic effects of opioid analgesia in the treatment of severe abdominal pain in kids and adolescents.

**Methods:**

A comprehensive electronic search will be done on Web of Science, EMBASE, PubMed, WanFang database, Chinese National Knowledge Infrastructure, and the Cochrane Library from their establishment to May 2021. The search will identify and retrieve all randomized controlled trials that describe the clinical therapeutic effects of opioid analgesia to treat severe abdominal pain in adolescents and children. Two independent authors will shortlist studies that meet the inclusion criteria, extract data from selected studies, and evaluate the risk associated with bias in the selected articles. We will use RevMan (v: 5.3) to conduct all the data synthesis.

**Results:**

This meta-analysis will conduct a high-quality synthesis on present evidence related to the usage of opioid analgesia to treat severe abdominal pain in both kids and adolescents.

**Conclusion:**

Our findings will summarize the present evidence and help judge whether opioid analgesia is an effective and safe line of treatment for severe abdominal pain.

**Ethics and dissemination:**

This study will use pre-published data, and as such, it does not require ethics approval.

**OSF registration number:**

May 29, 2021.osf.io/fp9ym (https://osf.io/fp9ym/).

## 1 Introduction

Acute abdominal pain is a commonly reported physical manifestation among patients who visit the emergency department, accounting for nearly 6% to 10%of all emergency ward calls, and these numbers continue to rise.^[1–3]^ Generally, a person is kept in emergency care wards for >6 hours, and admission rates are almost 25%.^[1,2]^ Historically, clinicians have been reluctant to administer analgesia to inpatients who report severe abdominal pains, primarily due to the widespread belief of management concerns.^[4–7]^ Certainly, pediatric emergentologists have acknowledged that discontentment from surgical professionals has obstructed the administering of analgesia.^[7]^ In spite of increasing evidence over a decade pointing out there is no association between opioid analgesia and an elevated risk of diagnostic or management errors, it remains a prevalent phenomenon.^[8,9]^

In ICUs and pre-hospital settings, opioids are commonly used as analgesics to provide relief to those suffering from severe abdominal pain. Parenteral management of opioids is justifiable when admitted individuals are exhibiting severe abdominal pain needing urgent relief, or when the patient cannot ingest medicine orally. Despite several negative side-effects, such as decreased blood pressure, depression, vomiting, and nausea, opioids are mostly safe when managed properly.^[4,6,10]^ Reportedly, it is safe to use opioids to treat patients suffering from severe abdominal pain without a risk of obscuring the diagnosis. However, most physicians are reluctant to administer opioid analgesia under such circumstances.^[4,11]^ Therefore, the present study will summarize the clinically critical outcomes of analgesic efficacy and safety of opioid analgesia to treat severe abdominal pain in kids and adolescents.

## 2 Methods

This study will be conducted in accordance with the guidelines outlined in the Preferred Reporting Items for Systematic Review and Meta-analysis Protocols (PRISMA-P) statement. The present study is registered on the OSF (http://osf.io/) with registration DOI number 10.17605/OSF.IO/FP9YM.

## 3 Inclusion criteria for study selection

### 3.1 Types of studies

The present meta-analysis only includes randomized controlled trials that evaluate the efficacy and safety of using opioid analgesia to treat severe abdominal pains in kids and adolescents.

### 3.2 Types of participants

Both minors and adult participants diagnosed with acute abdominal pain will be included regardless of the sex, race, and country.

### 3.3 Types of interventions

The intervention set must have received opioid analgesia treatment. The control set will receive any other treatment, expect for opioid analgesia.

### 3.4 Types of outcome measures

The primary outcomes are the difference in the dynamic of self-reported pre- and postintervention pain scores between opioid analgesia and control sets and rate of accurate management decisions. The minor outcomes include variation in the severity of the pain, fluctuations in the physical exploration, incorrect diagnosis, morbidity, and opioid-related side effects.

## 4 Search methods for identification of studies

### 4.1 Electronic searches

A comprehensive electronic search will be done on Web of Science, EMBASE, PubMed, WanFang database, Chinese National Knowledge Infrastructure, and the Cochrane Library from their establishment to May 2021. The search will identify and retrieve all randomized controlled trials that describe the clinical therapeutic effects of opioid analgesia to treat severe abdominal pain in adolescents and children. We will use a combination of the following terms to search above-mentioned databases: “abdominal pain,” analgesi&ast;, and “randomized controlled trial.”

### 4.2 Searching other resources

The present protocol will also include other related studies identified by searching articles from ClinicalTrials.gov (http://clinicaltrials.gov) and gray literature.

## 5 Data collection and analysis

### 5.1 Selection of studies

**Figure 1:**
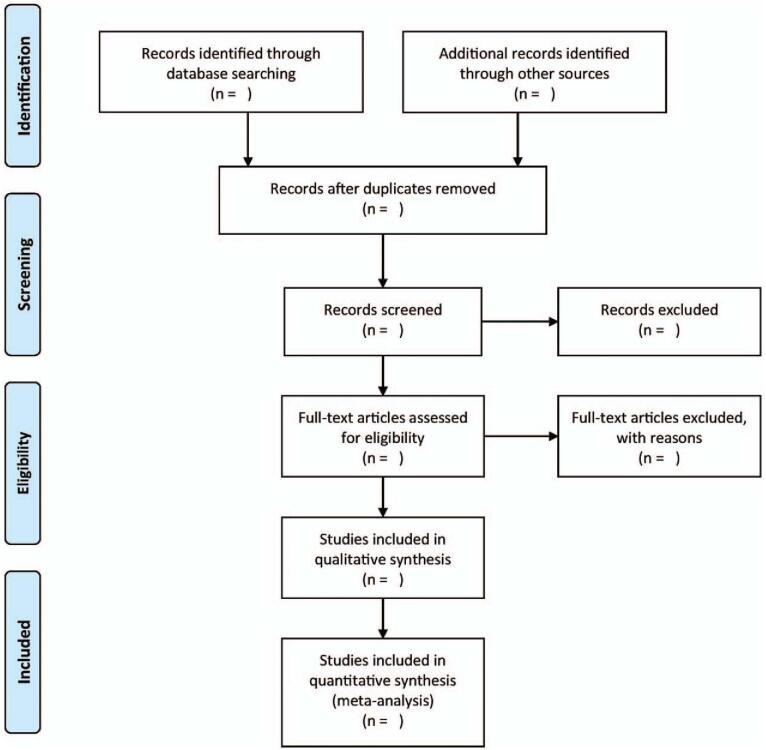
Flowchart of study selection process.

A pair of autonomous authors is needed to screen and select eligible studies. In brief, they will be screening the titles/abstracts to reject duplications and studies that fail to satisfy the criteria for inclusion. Finally, the studies to include shall be decided once the authors scrutinize the complete text of the studies. All disagreements will be arbitrated through consultation with a third independent author. The complete study selection process is illustrated in Figure 1.

### 5.2 Data extraction and management

A pair of autonomous authors will use a pre-specified data acquisition table to extract the following data: basic information (author, title, publication date, ethnicity, and country), study design (size of the samples, randomization details, allocation, binding methods, intervention approaches, and duration), and outcome measures. All disagreements will be arbitrated via consultation with an additional independent author.

### 5.3 Assessment of risk bias

A couple of independent authors will utilize the Cochrane Collaboration Tool to evaluate the risk of bias in the included articles across 6 domains, namely order generation, concealment of allocation, blinding, incomplete/missing outcomes, selective reporting bias, and extra bias.^[12]^ All disagreements will be arbitrated via consultation with an additional independent author.

### 5.4 Measures of treatment effect

This study will use a relative risk with 95% confidence intervals for dichotomous data, while we will use mean differences or standardized mean differences with 95% confidence intervals for continuous data.

### 5.5 Assessment of heterogeneity

The *χ*^2^ test and *I*^2^ statistic shall be used to evaluate the statistical heterogeneity. In the case of substantial heterogeneity (*P* < .1 or I^2^ > 50%), we will use the random-effects model. In the case when the heterogeneity is low (*P* > .1 or *I*^2^ < 50%), we will apply the fixed-effects model.^[13,14]^

### 5.6 Assessment of reporting biases

We will make use of Funnel plots to investigate possible bias in reporting if at least ten trials are selected for inclusion. Additionally, the Egger regression test shall also be used to categorize the asymmetry in funnel plots.

### 5.7 Assessment of reporting biases

We will conduct a sensitivity analysis to investigate the robust level in the pooled effects of our findings.

## 6 Discussion

This study will analyze the efficacy and safety of opioid analgesia to treat severe abdominal pain in kids and adolescents using a structured and valid methodology. To the best knowledge of the authors, there has been no previous systematic review to address the issues related to the appropriateness of such treatments. Studies evaluating analgesic efficacy and safety of opioid analgesia to treat severe abdominal pain in kids and adolescents are limited, and the results remain controversial. Therefore, we will conduct this study to provide a summary of all the clinically crucial results related to the analgesic efficacy and safety of using opioid analgesia to alleviate the adverse effects of severe abdominal pain in kids and adolescents. Conclusions drawn from the present meta-analysis will provide helpful information for clinical practitioners, scholars, patients, policymakers, and investigators when making decisions.

## Author contributions

**Conceptualization:** Jie Li, Fei-Yan Hu.

**Data curation:** Jie Li.

**Formal analysis:** Jie Li.

**Investigation:** Jie Li, Fei-Yan Hu.

**Methodology:** Fei-Yan Hu, Guo Zhong.

**Project administration:** Fei-Yan Hu, Guo Zhong.

**Resources:** Fei-Yan Hu, Guo Zhong.

**Software:** Jie Li.

**Supervision:** Fei-Yan Hu.

**Validation:** Jie Li, Guo Zhong.

**Visualization:** Jie Li, Fei-Yan Hu, Guo Zhong.

**Writing – original draft:** Jie Li, Fei-Yan Hu.

**Writing – review & editing:** Guo Zhong.
